# Current status of biomarker research in neurology

**DOI:** 10.1186/s13167-016-0063-5

**Published:** 2016-07-04

**Authors:** Jiri Polivka, Jiri Polivka, Kristyna Krakorova, Marek Peterka, Ondrej Topolcan

**Affiliations:** 1 Department of Neurology, Faculty of Medicine in Plzen, Charles University Prague, Husova 3, 301 66 Plzen, Czech Republic; 2 Department of Neurology, Faculty Hospital Plzen, E. Benese 13, 305 99 Plzen, Czech Republic; 3 Department of Histology and Embryology, Charles University Prague, Husova 3, 301 66 Plzen, Czech Republic; 4 Biomedical Centre, Faculty of Medicine in Plzen, Charles University Prague, Husova 3, 301 66 Plzen, Czech Republic; 5 Central Imunoanalytical Laboratory, Faculty Hospital Plzen, E. Benese 13, 305 99 Plzen, Czech Republic

**Keywords:** Biomarker, Personalized medicine, Neuro-oncology, Stroke, Alzheimer’s disease, Parkinson’s disease, Multiple sclerosis, Predictive preventive personalized medicine

## Abstract

Neurology is one of the typical disciplines where personalized medicine has been recently becoming an important part of clinical practice. In this article, the brief overview and a number of examples of the use of biomarkers and personalized medicine in neurology are described. The various issues in neurology are described in relation to the personalized medicine and diagnostic, prognostic as well as predictive blood and cerebrospinal fluid biomarkers. Such neurological domains discussed in this work are neuro-oncology and primary brain tumors glioblastoma and oligodendroglioma, cerebrovascular diseases focusing on stroke, neurodegenerative disorders especially Alzheimer’s and Parkinson’s diseases and demyelinating diseases such as multiple sclerosis. Actual state of the art and future perspectives in diagnostics and personalized treatment in diverse domains of neurology are given.

## Background

The term “personalized medicine” (PM) was first explained in detail in Kewal K. Jain’s Textbook of Personalized Medicine, published in 1998. The first reference made to PM in the MEDLINE database dates back to 2000. It describes the predicted effect of albuterol in asthma sufferers based on their DNA makeup. This was the first example of personalized treatment based on human genome sequencing [1]. Personalized medicine is closely related to pharmacogenetics and pharmacogenomics, and the field primarily grew in the period after the complete human genome was mapped in 2000 [2].

It is difficult to offer a precise definition of personalized medicine. Sometimes other terms are used, such as therapy according to diagnosis, genomic medicine, genotype-based therapy, individualized or individual medicine, omics-based medicine, predictive medicine, rational drug selection, and tailored therapy.

In this review, we provide the overview and a number of examples where personalized medicine, or an individualized approach to therapy, has been recently applied in neurology domain. Our article conforms with the recommendations of the “EPMA White Paper” [3].

## Biomarkers and personalized medicine in neuro-oncology

In recent times, there has been a significant expansion of knowledge in the field of neuro-oncology regarding the onset and development of neoplastic disease at the genomic and epigenomic levels. New prognostic and predictive biomarkers for the disease are appearing and the basic view of the histological typing of central nervous system (CNS) tumors is changing. In the near future, it will likely be necessary to integrate personalized medicine into standard clinical care for patients suffering from neurological cancer. The current World Health Organization (WHO) typing from 2007 recognizes more than 130 different histopathological units of primary CNS tumors [4]. This represents a very extensive and markedly heterogeneous group of diseases, with individual types of tumors exhibiting various biological behaviors. Moreover, even in the given histopathological units, further segmentation is starting to establish itself that is based on molecular genetics profiles resulting from international groups’ current whole exome and whole genome sequencing studies, which focus on the genomics and epigenomics of neoplastic diseases. An ambitious project that may serve as an example is a tumor atlas of selected cancer diagnoses, The Cancer Genome Atlas (TCGA), sponsored by the National Institutes of Health (NIH) in the United States. In a sample of 500 previously untreated patients, the NIH was the first in the world to clarify changes in the most frequent and most malignant primary brain tumor, glioblastoma multiforme (GBM), at the DNA, mRNA and short non-coding microRNA’s levels [5]. This led to the new division of what till then had been a homogenous group, GBM, into four subtypes according to dissimilar gene expression profiles with differing responses to conventional chemotherapy. In the future, this may contribute to the further personalization of tumor therapy for this type of disease. Despite the marked diversity of primary CNS tumors, the absolute majority are tumors of neuroepithelial tissue, specifically the astrocytoma group. It is further divided according to growing malignancy potential into four groups of gliomas, with GBM having the highest representation. Despite the limited options for choosing standard glioma therapy for now, new prognostic and predictive biomarkers have recently appeared that will soon allow for therapy to be “tailored” to each patient with the aim of achieving longer survival and better quality of life [6, 7].

The forecast of a more favorable prognosis as well as the prediction of a better response to the therapy administered are both important elements in the basic principles of personalized medicine. In this regard, several predictive CNS tumor biomarkers are important and it is expected that they will be included in clinical practice. The predictive biomarker in GBM patients probably closest to practice is the status of O6-methylguanine-DNA methyltransferase (MGMT) promoter methylation [8–10]. The MGMT enzyme is able to effectively repair the DNA damage caused by temozolomide, a standard chemotherapy administered to patients. MGMT-promoter methylation reduces the production of the active enzyme and the patient’s response to temozolomide therapy is higher, as has also been reflected in the longer overall survival periods of GMB patients at 21.7 vs. 15.3 months [11]. The status of MGMT-promoter methylation may also serve as a predictive biomarker in relation to radiotherapy [12].

The isocitrate dehydrogenases (IDH) mutation is an important glioma biomarker near clinical application that is able to contribute to determining the patient’s prognosis. IDH is an important Krebs cycle enzyme and has three different isoforms—IDH1 (found in the cytoplasm and peroxisomes) and IDH2 and 3 (in the mitochondria) [13]. Recurrent mutations in IDH were first systematically described in patients with GBM, though only in about 5 % of the patients [14]. In contrast, gene mutations for IDH1 and IDH2 were found with high frequency in diffuse astrocytomas (70–80 %) and anaplastic astrocytomas (up to 50 %) [15]. Mutations in IDH1 show conservative substitution of R132H in 90 %; R132C, R132G, R132S and R132L are also known. Mutations in IDH2 are far more rare and primarily involve R172 substitution [16]. In terms of personalized medicine in neurological cancer patients, the marked impact of these mutations on the disease prognosis is especially important, regardless of the therapy used. It has been found that GBM patients with IDH1/2 mutations have a significantly longer median of overall survival than patients without these mutations. Several different papers have shown 3.8 vs. 1.1 years, 2.6 vs. 1.3 years, 2.3 vs. 1.2 years, and 3 vs. 1 year of overall survival [14, 15, 17–20]. Even more significant differences in overall survival were found in patients with anaplastic astrocytomas: 5.4 vs. 1.7 years, 6.8 vs. 1.6 years and 7 vs. 2 years [15, 17, 18]. Similarly, diffuse astrocytoma has a far better prognosis if there is a mutation in IDH1/2: 12.6 vs. 5.5 years [17]. Recent meta-analysis of 55 observational studies has shown that patients with gliomas positive for IDH1/2 mutations have improved both overall survival and progression-free survival, especially patients with WHO grade III and grade II-III tumors [21]. Moreover, the combination of two biomarkers (IDH1 mutation and MGMT methylation status) outperforms either IDH1 mutations or MGMT methylation alone in predicting survival of glioblastoma patients [22].

Oligodendrogliomas are also important representatives of neuroepithelial tumors of the CNS. WHO grade III anaplastic oligodendrogliomas (AO) are among those with a higher malignancy potential [4, 23]. The median overall survival of AO patients is reported as between 2 and 6 years with standard treatment. Conventional radiotherapy may be augmented with a combination regimen of PCV (procarbazine, lomustine and vincristine) chemotherapy, though the effect of combined radiotherapy and chemotherapy on the overall survival of newly diagnosed AO patients has not been sufficiently proven for a non-selected population [24]. A certain breakthrough in regards to adjuvant chemotherapy in the treatment of AO occurs only with the application of the principles of personalized medicine and predictive biomarkers. Molecular changes in a certain group of AOs, the co-deletion of the short arm of chromosome 1 (1p), and the long arm of chromosome 19 (19q) in neoplastic tissue, have been known for a relatively long time [25, 26]. Following several dramatic responses to AO therapy with a combination PVC regimen and radiotherapy in the 1990s, two international phase III clinical trials of combination chemo-radiotherapy for AO patients were launched: Radiation Therapy Oncology Group (RTOG) trial 9402 and European Organization for Research and Treatment of Cancer (EORTC) trial 26951. In these trials, the co-deletion of 1p/19q was monitored as a potential predictive biomarker of response to treatment. An ongoing analysis of the results of both studies in 2006 did not find a statistically significant relationship between the overall survival of patients who received radiotherapy alone or radiotherapy in combination with PCV and the presence of 1p/19q co-deletion [27, 28]. However, data from the long-term monitoring of both independent studies now clearly show a significant increase in the overall survival of patients with proven 1p/19q co-deletion in the neoplastic genome that were treated with combined radiotherapy and PCV chemotherapy. With a median patient monitoring period of 11.3 and 11.7 years in RTOG 9402 and EORTC 26951, respectively, the increase in the overall survival of AO patients was found to be 14.7 vs. 7.3 years (HR = 0.47, *P* < 0001) and NR (median overall survival not reached) vs. 9.3 years (HR = 0.56, *P* = 0.0594) for patients with 1p/19q co-deletion who received combined therapy [28, 29]. This clinical trial clearly demonstrates the predictive significance of the 1p/19q co-deletion biomarker in newly diagnosed AO patients and its effect on long-term survival for decades from the start of combined therapy.

These clinically very significant findings are successful examples of the integration of the principles of personalized medicine into modern neuro-oncology and will certainly soon become an important addition to standards in decision algorithms regarding care for these patients [10, 30–32] (Table 1).

**Table 1:** Examples of molecular biomarkers in gliomas and their clinical relevance

Molecular biomarker	Assessment method	Biomarker relevance		
		Diffuse gliomas (grade II)	Anaplastic gliomas (grade III)	Glioblastoma (grade IV)
1p/19q co-deletion	FISH, PCR	Positively prognostic	Positively prognostic for RT or CHT Predictive for PCV and RT	Very rare, unclear
*IDH1/2* mutations	RT-PCR, IHC, sequencing	Positively prognostic	Positively prognostic	Positively prognostic, rare Distinguishing secondary GBM
*MGMT* promoter methylation	Methylation-specific PCR	Unclear	Positively prognostic	Predictive for temozolomide
G-CIMP	Methylation-specific PCR	Positively prognostic	Positively prognostic	Positively prognostic

*Abbreviations*: *IDH1/2* Isocitrate dehydrogenase 1 and 2, *MGMT O6* methylguanine, *DNA* methyltransferase, *G-CIMP* Hypermethylator phenotype of cytosine-phosphate-guanine islands in gliomas genome, *GBM* Glioblastoma multiforme, RT Radiotherapy, CHT Chemotherapy, FISH Fluorescent in situ hybridization, RT-PCR Real time polymerase chain reaction, IHC Immunohistochemistry

## Biomarkers and personalized medicine in cerebrovascular diseases

Care for patients suffering from cerebrovascular diseases is highly sophisticated, based on high-quality diagnostics that allow physicians to determine the cause and extent of stroke and select the optimal treatment. In addition to clinical examinations and basic laboratory parameters, imaging methods (CT, CT perfusion, CT angiography, or MRI) are needed. In cases of acute cerebrovascular accidents, the rapid administration of the target treatment is essential to success. What else can biomarkers and concept of personalized medicine offer to this field?

The determination of blood biomarkers is an area that offers promise but as yet little applicability [33–35]. The ideal blood biomarker should be highly specific and sensitive, able to differentiate stroke mimics, determine the type and extent of stroke and have a predictive value for serious stroke complications, such as the risk of malignant edema or risk of hemorrhagic transformation of ischemic stroke.

However, such a single biomarker does not exist. In spite of this, the field is being carefully studied and certain partial successes have been described. Ischemic and hemorrhagic stroke lead to rapid changes in the signaling pathways and metabolic processes. Brain damage, ischemic cascade, activation of the immune system and blood-brain barrier dysfunction lead to an expression of biomarkers and the possible detection of these markers in peripheral blood.

The ischemic cascade includes the activation of glia, oxidative stress, the release of inflammatory mediators and neuron damage [36]. Biomarkers with relative specificity towards these processes could be detected in blood [37]. Biomarkers for glial activation include S100 beta, glial fibrillary acidic protein and myelin basic protein. S100 beta is also marker of astrocyte activation and brain tissue injury with low specificity for ischemic stroke. Glial fibrillary acidic protein differed in hemorrhagic stroke compared with ischemic stroke (*p* < 0.0001) within 4.5 h of symptom onset [38]. Myelin basic protein is one of the main component of CNS myelin and could be found in cerebrospinal fluid (CSF) and blood within first hours after stroke onset. Determination in blood is sufficient. The release of these biomarkers after stroke is associated with the volume of brain lesions. PARK-7 and malondialdehyde are biomarkers of oxidative stress. Their potential clinical application is in early diagnosis of stroke and in prediction of stroke prognosis. Biomarkers of inflammation include C-reactive protein, matrix metalloproteinase (MMP) 9, interleukin 6 (IL-6) and tumor necrosis factor alpha (TNF-alpha). Namely MMP 9 have been widely investigated for its role in disruption of the blood-brain barrier and extracellular matrix following stroke [37, 39].

The main biomarkers of neuronal damage are neuron specific enolase and *N*-methyl-d-aspartate receptor (NMDA-R). d-dimmer, fibrinogen, fibronectin, von Willebrand factor and thrombomodulin are biomarkers of endothelial dysfunction. Additional biomarkers are lipoprotein-associated phospholipase A2 and brain natriuretic peptide (BNP). Blood biomarkers can help in distinguishing the etiology of stroke (BNP in cardioembolic stroke), in predicting early neurological deterioration and clinical outcome (S100 beta, MMP, IL-6, TNF-alpha), and in predicting hemorrhagic transformation (cellular fibronectin, MMP 9) [37, 40, 41]. However, most of these biomarkers do not have a sufficient level of sensitivity, specificity or both. Moreover the heterogeneity of different cell populations in the brain and their ischemia tolerance and distributions within the central nervous system, the complexity of the ischemic cascade, and presence of the blood-brain barrier cause that no single biomarker has ever been demonstrated to be clinically useful. For this reason, batteries of biomarkers are described that offer greater predictive value when applied. For example, a panel of biomarkers for the ischemic cascade can distinguish patients with acute stroke from age and gender-matched control subjects with a sensitivity and specificity of 90 % [42].

Another prospect is the use of biomarkers that signify changes in the gene expressions that occur in minutes and hours after the onset of stroke. These include capturing changes in certain mRNA in the peripheral blood and circulating leukocytes [43] or determining several circulating microRNA [44, 45].

The clinical application of blood and gene biomarkers in the acute phase of stroke has thus far run up against technical limitations, speed of detection and especially high cost. However, they may offer valuable additional information about the type and prognosis of stroke.

Biomarkers can also be used in the field of stroke prevention. Clopidogrel is transformed into an active metabolite with a significant anti-platelet effect by cytochrome P-450. Carriers of at least one of the transformed allele of the enzyme CYP2C19 (about 30 % of the population) have an increased risk of vascular accidents. In the TRITON TIMI-38 study, they had a 53 % greater risk of stroke, heart attack, and cardiovascular death when treated with clopidogrel [46]. Dicumarol is used for a 30 % reduction in the relative risk of cardioembolic stroke. Its individually transformed effect is tied to polymorphisms in the genes VKORC1 and CYP2C9 [47, 48]. Statins are used for the relative reduction of around 20 % in the onset of stroke. Statin-induced myopathy is a risk associated with their use. This effect is tied to rs4149056 polymorphism in the SLCO1B1 gene located on chromosome 12. Persons with one variant allele have a 4.5 times greater risk of statin-induced myopathy. Homozygotes with both variant allele (2.1 % of the investigated population) have as much as a 17-times greater risk of statin-induced myopathy [49].

## Biomarkers and personalized medicine in neurodegenerative diseases

Biomarker research in neurodegenerative disease is a rapidly advancing area in personalized medicine. The good biomarker should have specificity more than 80 % and the same level of sensitivity (more than 80 %). The role of these markers is not only diagnostic; they have also prognostic potential or role in development of new treatment [50, 51]. A large number of molecules have been evaluated and associated with different neurodegenerative disorders, but only several of them are validated and well-established. Current status of the development of new biochemical biomarkers for Alzheimer’s disease and Parkinson’s disease, two most common neurodegenerative disorders, is discussed.

### Alzheimer’s disease

Alzheimer’s disease (AD) is the most common neurodegenerative disorder with prevalence from 2 % in seventh decade to 25 % in ninth decade of life [52]. Diagnosis is difficult especially at early stages before all of sings meeting criteria of AD are presented. So, there is a great field for exploration of novel specific and sensitive biochemical markers which do not constitute discomfort for the patient and which are cost-effective. Useful candidates have been found in blood and in CSF [53]. Potential and already used biomarkers of AD can be divided according to assumed mechanisms of pathogenesis into markers related to the amyloidogenic pathway and cholesterol metabolism, markers of oxidation, markers of immunologic mechanism and inflammation, markers associated with microvascular changes and proteome-based plasma biomarkers [54].

Major CSF biomarkers that are used in clinical practice are tau proteins (T-tau, P-tau) and amyloid β (especially Aβ40, Aβ42). Amount of T-tau correlates with the intensity of neuroaxonal degeneration, level of P-tau reflects tangle pathology and Aβ correlates with plaque pathology [55–57]. The specificity and sensitivity of these biomarkers is between 80 and 90 % [58]. However, the lumbar puncture is relatively invasive practice, especially repeated and in elderly patients. More comfortable tests are searched especially in blood and plasma.

Several studies deal with antibodies against amyloid β as a biomarker of AD. Du at al. describe significantly lower titres of Aβ antibodies in patients with AD [59], but not in another study [60]. It was hypothesized that more relevant target provides detection of low molecular weight oligomeric cross linked Aβ protein species (CAPS) and anti-CAPS antibodies [61]. Anti-CAPS are significantly reduced in AD patients. These results suggest possibility of using anti-CAPS as a plasma biomarker of AD and promising possibility of therapeutic use in the future.

Further very interesting results provides a research of amyloid precursor protein (APP). This protein is present in central nervous system, but it is also expressed in peripheral tissues such as in circulating cells. The isoforms of APP can be detected in platelets membrane. The intact 150 kDa weight APP is divided into two forms after platelet activation [62]. The ratio of forms with molecular weight 120–130 kDa and of 110 kDa weight are called “platelet APP isoform ratio,” and it is decreased in AD and mild cognitive impairment (MCI) not in other dementias [63, 64].

Markers related to cholesterol metabolism are total cholesterol plasma level, CSF and plasma level of 24S-hydroxycholesterol, plasma level of apolipoprotein E and apolipoprotein E genotype. There are three major human apolipoprotein E isoforms—ε2, ε3, and ε4; they are encoded by different alleles with different risk for development of the AD [65–67]. These all provide different and ambiguous results and the interpretation for clinical use remains to be clarified in future studies [68]. The promising biomarker would be 24S-hydroxycholesterol that is elevated in AD patients’ CSF and plasma [69]. New studies demonstrate a sensitive and a powerful specific biomarker for early and easy AD diagnosis—desmosterol. Desmosterol was found to be decreased in AD plasma versus controls and more significant in females [70].

Promising but also inconsistent results provided studies of oxidation and immunologic biomarkers. AD and vascular dementia are associated with decrease of plasma and serum levels of vitamins A, C, E, and dietary intakes of the three antioxidants can lower the risk of AD [71]. Sano et al. found that supplementation of vitamin E delayed progression of AD [72] and elevated levels of tocopherol and tocotrienol forms are associated with reduced risk of cognitive impairment in older adults [73]. A significant association between AD and low levels of vitamin D has been demonstrated [74]. Plasma level of isoprostane 8,12-iso-iPF2α-VI as a specific and sensitive marker of lipid oxidation is increased in AD and correlates with level of cognitive and activities of daily living impairment [75]. Other results show that plasma or urine level of this marker do not accurately reflect situation in the central nervous system [76]. Controversial data have been published about α1—Antichymotrypsin (ACT)—which is one of the components of senile plaque. High plasma levels of ACT would be associated with an increased risk of AD [77].

The next candidate biomarker is Alzheimer-associated neuronal thread protein (AD7c-NTP) which can be detected in CSF, brain-tissue extracts, cortical neurons, and urine. Its level also positively correlated with degree of dementia [78].

Neuroimaging techniques can disclose signature abnormalities of brain morphology and function many years before AD symptoms appear. A number of neuroimaging candidate markers are promising, such as hippocampus, amygdala, and entorhinal cortex volumes, basal forebrain nuclei or atrophy of the grey matter of the medial temporal and dorsolateral frontal lobes [58, 79, 80]. Sabuncu et al. examined a total of 317 participants with baseline cerebrospinal fluid biomarker measurements and 3 T1-weighted magnetic resonance images obtained within 1 year. Their results show that AD-specific cortical thinning and hippocampal volume loss are consistent with a sigmoidal pattern, with an acceleration phase during the early stages of the disease [81]. Fluorodeoxyglucose positron emission tomography has shown a specific pattern of regional hypometabolism. Hippocampal glucose metabolism reduction was found in both mild cognitive impairment and Alzheimer disease and contributes to their diagnostic classification [82, 83]. Fleisher et al. used positron emission tomography (PET) and florbetapir F18 to image cortical amyloid in patients with mild cognitive impairment or dementia due to Alzheimer disease. Their analysis confirmed the ability of florbetapir-PET to characterize amyloid levels in clinically probable AD and mild cognitive impairment [84, 85].

### Parkinson’s disease

Parkinson’s disease (PD) is another most common neurodegenerative disease in human population with prevalence of about 1 % after the sixth decade. It is expected a doubling of prevalence until 2030 [86]. Cardinal motor symptoms of the disease (tremor at rest, rigidity, bradykinesia, and postural instability) are presented after more than 50 % of dopaminergic nigral cells are damaged [87]. PD is not just motor disorder and its non-motor symptoms often precede the motor ones. These premotor markers include olfactory and autonomic dysfunction, sleep disorder, depression, and cognitive disturbances [88, 89]. Biomarkers of PD are required for detection persons at risk, for recognition of PD before clinical symptoms are presented, prediction of disease progression, for stratification of success of treatment or for distinguishing PD from parkinsonism. Unfortunately, no validated diagnostic biomarker of PD is available.

Similar to AD, perspective biomarkers of PD can be divided into biomarkers belonging to oxidative stress, dopamine metabolism, α synuclein, auto antibodies against α synuclein and inflammatory markers. Novel approach is also demonstrated by research in the field of metabolomic profiling.

The most promising results are provided by the research of α synuclein which is one of the main component of Lewy bodies and has been detected in serum, plasma, saliva and CSF [86]. Studies of Mollenhauer and Devic showed decreased level of α synuclein in CFS in PD and in parkinsonism [90, 91]. Measurements of α synuclein and phosphorylated α synuclein concentrations can distinguish PD from multiple system atrophy (MSA) and progressive supranuclear palsy (PSP) [92]. MSA is a rare neurodegenerative disorder previously called Shy-Drager syndrome. It is classified into two types: parkinsonian and cerebellar phenotypes. It is characterized by abnormal accumulation of α-synuclein but in contrast to PD with mainly accumulation in glial cytoplasmic inclusions [93]. And PSP is a neurodegenerative syndrome that is clinically characterized by progressive postural instability, supranuclear gaze palsy, parkinsonism, and cognitive decline [94]. El Agnaf reported that oligomeric soluble forms of α synuclein are significantly elevated in plasma of PD patients [95]. Also auto antibodies against α synuclein are elevated in 90 % of familiar PD cases and 51 % sporadic cases [96].

Several studies show abnormalities of inflammatory markers. Chen reported that higher level of IL-6 is associated with greater risk of PD [97] and Scalzo found that higher levels of soluble TNF receptor-1 are connected with early onset of disease [98]. Interesting role in pathogenesis of PD plays increased oxidative stress. For example, significant reductions of mitochondrial complex I was found in platelets membrane in PD patients [99] but these results were not confirmed in another study [100]. Coenzyme Q10 (CoQ10) related to PD is also studied. Platelet CoQ10 redox ratio (reduced CoQ10 to oxidized CoQ10) was significantly decreased in de novo PD patients. Redox ratio was not correlated to disease severity [101]. Schwarzschild reported that high level of serum urate is connected with slower progression of PD and therefore urate is the first molecular factor linked directly to the progression of typical PD [102].

Recent studies in personalized medicine of neurodegenerative diseases are focused on metabolomic biomarkers. That means identification and quantification of intracellular metabolites, small changes in mRNA and exploration of small molecules in tissues, cells and body fluid that can be significant for specific disease or process including PD. A lot of another studies investigate a role of different molecules in PD. Chen discovered that low level of epidermal growth factor in plasma is linked with cognitive function and can be used as a marker of cognitive decline in patients with PD [103].

Over 25 genetic factors have also been shown to constitute risk factor for PD [104]. For example, homozygous and heterozygous mutations of the glucocerebrosidase gene are a major risk factor for PD [105]. The mutations in the gene for α-synuclein in familial forms of Parkinson’s disease have led to the belief that this protein has a central role and is associated with more rapid disease progression; dementia or hallucinations [106]. Newly Azuma et al. reported mutation of the cyclic nucleotide phosphodiesterase 8B gene as one of the causal gene mutation of this disease [107].

Magnetic resonance imaging (MRI) positron emission tomography, transcranial sonography or single-photon emission tomography (SPECT) allow the non-invasive tracking of molecular targets of relevance to neurodegeneration [108]. MRI can provide information about disease-induced changes in the structure and nigral abnormalities and about reduction of brain regional *N*-acetyl-aspartate that is biomarker of neuronal loss [109, 110]. Fibrillar amyloid load can be quantitfied in vivo with PET [111]. Next PET biomarkers include e.g., F-18 fluorodeoxyglucose uptake for mitochondrial bioenergetics [112, 113], F-18 DOPA uptake which is associated with an increased risk for later motor complications and comprises a disease-intrinsic predisposing factor for their development [114] or a dopamine transporter marker [(11) C] CFT and [(11) C] (R)-PK11195 to investigate changes in microglial activity [115]. Siderowf et al. tried to evaluate the relationship between [99mTc] TRODAT-1 SPECT imaging, odor identification skills, and motor function in patients with early PD and they found that olfactory function is highly correlated with dopamine transporter imaging abnormalities [116] and also that it correlated with anxiety and depression symptoms [117]. Impulse control disorders including compulsive gambling, buying, eating, and hypersexuality are relatively frequent especially in younger male PD patients, especially in those treated with dopamine agonists. It may cause catastrophic consequences, including financial ruin, divorces, loss of employment, and others. Pharmacological treatment should be individualized based on patient’s unique neuropsychiatric profile, social support, medical comorbidities, tolerability, and motor symptoms [118, 119].

Several markers have shown the potential of effective biomarkers but they need verification in further studies. It is likely that a single measure and one biomarker are not sufficient and that only combination of various biomarkers as well as the clinical relevant patient characteristics can provide complex and useful information on disease.

## Biomarkers and personalized medicine in demyelinating diseases

The current interest in the field of demyelinating disease focuses on multiple sclerosis (MS), not just for its frequency of occurrence, but also because it is a disease that disables young working-age population.

Recently, the diagnosis of this disease was very carefully worked up and also simplified, especially through the use of MRI. The MRI of brain and spinal cord is currently used as the main supporting diagnostic method [120, 121]. Among other supportive parameters in MS diagnosing belongs the testing for cerebrospinal fluid-restricted oligoclonal bands (OCB) by isoelectric focusing, which is used to detect intrathecally produced total IgG. Another characteristic findings in patients with MS is the polyspecific intrathecal B cell response against neurotropic viruses, specifically against measles virus, rubella virus, and varicella zoster virus, also known as an MRZ (Measles antibody index, Rubella antibody index, Zoster antibody index) reaction and abnormalities in visual, auditory, somatosensory, and motor-evoked potentials [122–126].

In the last few, years the treatment of MS achieved a huge progress with the arrival of disease-modifying drugs (interferons and glatiramer acetate, natalizumab or fingolimod and lately also alemtuzumab, dimethyl fumarate, teriflunomid). Moreover new oral and parenteral drugs are already on the verge of clinical use, which can bring more hope in the treatment of MS. Currently there is a number of drugs that differs in their efficiency and safety profile, due to this fact the problem is how to select patients according to their susceptibility to treatment with specific drug and how to prevent or minimize the adverse effects. The timing of the treatment is crucial for the patient’s prognosis. The best is to start when only clinically isolated syndrome (CIS) is present. But not only early treatment is important, huge role plays also the choice of the most suitable drug according to the clinical and MRI findings, the presence of underlying diseases and other related aspects. The aim is to stabilize the process of this disease and minimize the adverse effects. That is the goal of personalized medicine in patients with MS.

Personalized medicine in the field of MS is based on couple of aspects of the disease. These are demyelination and progression of inflammation, neurodegeneration (axonal loss), progression of disability, and therapeutical response. It is very important to keep all these aspects in mind when choosing the best therapy.

The key question seems to be how to determine the risk of conversion from clinically isolated syndrome to clinically definitive diagnosis of MS. The answer to this question brings the multicentre studies published in 2015. The results showed the higher risk of conversion in patients with the presence of OCB in CSF, higher number of lesions on MRI and younger age patients. Low level of vitamin D has also showed a small predictive value to conversion to clinically definite multiple sclerosis (CDMS), but this parameter is still the subject of investigation. On the other hand other observed parameters such as sex, smoking, CSF cytology, type of clinical presentation of CIS, the presence of IgG antibodies against EB virus or IgG antibodies against CMV, did not show any predictive value for conversion from CIS to CDMS. Multivariable regressive analysis has shown that accumulation of single risk parameters leads to increasing risk of conversion from CIS to CDMS and malignant course of diseases [127]. Another recent study dealt with similar topic, specifically focusing on predictive factors for conversion from CIS to CDMS. The results came out of long term data collection already since 1995. Clinical status of the patients was thoroughly examined in the interval from 3 to 6 months and brain MRI was done after 12 months and then every 5 years. Based on this analysis the risk variables were established for developing CDMS and expanded disability status scale (EDSS) 3.0—the count of lesions on brain MRI, the presence of oligoclonal bands in CSF, type of clinical presentation of CIS, sex, and age. Thanks to all these variables it was possible to analyse the risk of developing CDMS or risk of reaching EDSS 3.0 for every patient with CIS. It is a very dynamic model, which is able to valorize the risk again after 12 months based on the presence of relapses, new T2 lesions on MRI and type of treatment in the last 12 months. Regarding all the results it is possible to re-analyze the risk of progression of the disease and therefore change the treatment if necessary [128].

Another biomarker that has been recently followed in patients with MS is vitamin D. Its role in bone metabolism and calcium homeostasis is already well-known, but recently it has been proved also its immunomodulatory, anti-inflammatory and neuroprotective effect. Therefore, many studies now focus on the influence of vitamin D to the development and course of autoimmune diseases such as MS. Many epidemiologic, preclinical and clinic data showed that low level of vitamin D had proven to be one of the risk factors in developing MS and is often linked with higher activity and progression of the disease [129–131]. In 2014, Kimbourgh et al. published the results of a study examining the risk factors of transversal myelitis reoccurring. Low level of vitamin D during the first attack of transversal myelitis was proven among the highest risk factors of developing another attack [132].

In 2013 a team Sormani published a new modified Rio score, which helps to identify patients with positive response to treatment with interferon beta. Those patients are called responders. This score analyses the presence of new T2-weighted lesions on MRI, the number of relapses after a 1 year of treatment with interferon beta. Based on those results patients are divided into three groups; first group involves patients with the lowest risk of progression of the disease and therefore patients with the best response to treatment—no relapse and max. five new T2-weighted lesions on MRI after 12 months of treatment. Patients with moderate risk of progression so-called partial responders belong to the second group. Those patients had only one relapse and max. five new lesion on MRI in the past year or they had no relapse at all but more than five new T2-weighted lesions on MRI. The last group contains patients with the highest risk of progression, so called non-responders to interferon beta, they showed more than two relapses in 1 year and max. five new lesions on MRI or 1 or 2 relapses and more than five new T2-weighted lesions on MRI. This scoring system comes from the original Rio et al. score published in 2009 with the addition of new parameter the progression of disability evaluated with EDSS [133, 134]. Stangel et al. suggested another scheme which includes more parameters that should be followed in patients with MS, regarding the aim of “no evidence of disease activity”. New parameters such as depression, anxiety, fatigue, quality of life and cognitive function were added to the already existing parameters (relapses, disability progression, and new lesions on MRI). It was proven as a very broad and sensitive tool, which helps to follow the disease progression even at the very beginning. These tools are nowadays very important not only for examining the stability of the disease but also for deciding about treatment escalation [135, 136].

The measuring of retinal nerve fibre layer thickness (RNFL) in the peripapilar area using the optical coherence tomography (OCT) is another very useful method with great potential. It is used for tracking the disability progression in patients with MS [137]. This method is non-invasive and can be relatively quickly and easily performed. Studies comparing the findings in the peripapilar area RNFL in patients with MS, neuromyelitis optica (NMO) and NMO spectrum disorders (NMOSD) showed a more severe infliction in patients with NMO and NMOSD. In patients with MS subclinical decrease of RNFL using the OCT can be found but this method still cannot be used as an independent method to differentiate MS and NMOSD in clinical praxis [138, 139]. One possible cause of not responding to treatment with interferon beta is the production of neutralizing antibodies (NAbs). These antibodies are bonded directly to the epitope of interferon beta and that disables its binding to the receptor. Up to 42 % of patients has shown the occurrence of these antibodies which usually form after 6 months of therapy. Their appearance after 2 years of therapy is very rare. NAbs are non-direct biomarkers and their presence only rises the possibility of decreased efficiency of interferon beta [140–142].

Commonly used direct biomarker in clinical practice is the production of MxA mRNA in patients treated with interferon. It is a protein produced by mononuclears due to stimulation of interferon protein. The evidence of MxA is based on determination of mRNA using PCR (polymerase chain reaction) method. Its transcription correlates with the efficiency of the drug [143–145].

Biomarkers that would currently seem as possible predictors of disease progression and that could warn against high risk of malignant course of disease are cerebral atrophy, atrophy of brain gray matter, diffusion tensor imaging (DTI) abnormalities, corpus callosum DTI abnormalities, upper cervical cord atrophy (UCCA), and early MR spectroscopy abnormalities. Based on the presence of these parameters the treatment of MS should be led the most effectively from the disease diagnosis [146–149].

Very crucial complication in using one of the most efficient drug natalizumab for treating the patients with MS is the occurrence of progressive multifocal leukoencephalopathy (PML). This important side-effect has appeared already during the treatment with other immunomodulatory drugs such as fingolimod and dimethyl fumarate, but now it is the center of attention in treatment with natalizumab. There are three main parameters to optimize the risk of PM occurrence—the duration of treatment, former immunomodulatory treatment, and seropositive tests to PML. There was an effort to find some other parameters, which could be used to select patients with low risk of PML during the treatment with natalizumab and also some parameters which could draw the attention to new or increasing risk during the treatment. One of the new parameters found is the antibody JCV (John Cunningham virus) index. The risk of PML increases with the increasing level of JCV antibodies. Also, antibodies seroconversion showed higher risk of PML occurrence. Another parameter is low count of T-lymphocytes expressing l-selectin (CD62L) which also leads to higher risk of PML appearance [150–153].

In last few years, the diagnosis of neuromyelitis optica drew big attention. The interest increased especially in 2004, when a highly specific serum antibody IgG was found. This antibody is aimed against aqua channel aquaporin 4 (AQP4) occurring especially in astrocytes. This key finding together with former findings of humoral pathogenic mechanisms led to distinguishing this diagnosis from MS even though the clinical picture and paraclinical findings often overlap. Sensitivity of this method is about 80 % combined with specificity reaching over 99 %. Couple recent studies have also proven the presence of antibodies against myelin oligodendrocyte glycoprotein (MOG-Ab) in patients with NMOSD. However, clinical meaning of these antibodies in the field of CNS demyelinating diseases remains uncertain. The highest profit is hoped to be in seronegative patients with NMOSD [154–157].

## Conclusions

The role of biomarkers and personalized medicine in neurology is becoming extensively important. The actual state of knowledge in several domains of neurology (neuro-oncology, cerebrovascular, neurodegenerative, and demyelinating diseases) was discussed in this article. A huge amount of perspective biomarkers could be routinely used in the neurological practice in many distinct settings. Especially in more precise diagnostics, better determination of patient prognosis or in prediction of treatment response. Future perspectives in neuro-oncology will bring the concurrent assessment of IDH1/2 mutations and MGMT promoter methylation status for glioblastoma and 1p/19q co-deletion for oligodendroglioma. In cerebrovascular diseases, the panels of blood biomarkers would be widely accessible and will serve especially for the outcome prediction. The anti-CAPS antibodies and β amyloid as well as amyloid precursor protein markers are promising in Alzheimer’s disease and α-synuclein in Parkinson’s disease. In demyelinating diseases, the goal for the future is to implement biomarkers that could help to distinguish patients with high risk of serious course from patients with potentially benign course of disease. Nevertheless, further validation of these biomarkers is necessary before their incorporation into standard clinical decision-making algorithms. Personalized medicine will certainly play the crucial role in the more effective, cheaper, and better tailored treatment of various neurological diseases in the near future.

## Abbreviations

*ACT*, α1-antichymotrypsin; *AD*, Alzheimer’s disease; *AD7c-NTP*, Alzheimer-associated neuronal thread protein; *AO*, anaplastic oligodendroglioma; *APP*, amyloid precursor protein; *AQP4*, aqua channel aquaporin 4; *BNP*, brain natriuretic peptide; *CAPS*, cross-linked Aβ protein species; *CIS*, clinically isolated syndrome; *CDMS*, clinically definite multiple sclerosis; *CNS*, central nervous system; *COX-1*, cyclooxygenase-1; *CSF*, cerebrospinal fluid; *CT*, computed tomography; *DTI*, diffusion tensor imaging; *EDSS*, expanded disability status scale; *EORTC*, European Organization for Research and Treatment of Cancer; *GBM*, glioblastoma multiforme; *IDH*, isocitrate dehydrogenases; *IL-6*, interleukin 6; *JCV*, John Cunningham virus; *MCI*, mild cognitive impairment; *MGMT*, o6-methylguanine-DNA methyltransferase; *MOG-Ab*, antibodies against myelin oligodendrocyte glycoprotein; *MRI*, magnetic resonance imaging; *MS*, multiple sclerosis; *Nabs*, neutralizing antibodies; *NIH*, National Institutes of Health; *NMDA-R, N*-methyl-d-aspartate receptor; *NMO*, neuromyelitis optica; *OCB*, oligoclonal bands; *OCT*, optical coherence tomography; *PCR*, polymerase chain reaction; *PCV*, procarbazine, lomustine, and vincristine; *PD*, Parkinson’s disease; *PM*, personalized medicine; *PML*, progressive multifocal leukoencephalopathy; *RNFL*, retinal nerve fiber layer thickness; *RTOG*, Radiation Therapy Oncology Group; *SNP*, single-nucleotide polymorphisms; *TCGA*, The Cancer Genome Atlas; *TNF-alpha*, tumor necrosis factor alpha; *UCCA*, upper cervical cord atrophy; *WHO*, World Health Organization
